# New Books

**Published:** 2010-09

**Biodata Mining and Visualiation: Novel Approaches**

Ilkka Havukkala

Hackensack, NJ:World Scientific, 2010. 324 pp. ISBN: 978-981-279-036-1, $88

**Certifiably Sustainable? The Role of Third-Party Certification Systems**

Committee on Certification of Sustainable Products and Services; National Research Council

Washington, DC:National Academies Press, 2010. 144 pp. ISBN: 978-0-309-14711-8, $35

**Climate Stabilization Targets: Emissions, Concentrations, and Impacts over Decades to Millennia**

Committee on Stabilization Targets for Atmospheric Greenhouse Gas Concentrations; National Research Council

Washington, DC:National Academies Press, 2010. 190 pp. ISBN: 978-0-309-15176-4, $45

**Computational Ecology: Artificial Neural Networks and Their Applications**

WenJun Zhang

Hackensack, NJ:World Scientific, 2010. 312 pp. ISBN: 978-981-4282-62-8, $96

**Enhancing Food Safety: The Role of the Food and Drug Administration**

Robert B. Wallace, Maria Oria, eds.

Washington, DC:National Academies Press, 2010. 520 pp. ISBN: 978-0-309-15273-0, $59

**Handbook of Climate Change and Agroecosystems: Impacts, Adaptation, and Mitigation**

Daniel Hillel, Cynthia Rosenzweig, eds.

Hackensack, NJ:World Scientific, 2010. 450 pp. ISBN: 978-1-84816-655-4, $168

**Health and Environment in Europe**

WHO Regional Office for Europe

Geneva:WHO Press, 2010. 156 pp. ISBN: 978-9-2890-4198-0, $40

**Household Use of Solid Fuels and High Temperature Frying**

IARC

Geneva:WHO Press, 2010. 400 pp. ISBN: 9789283212959, $55

**Informing an Effective Response to Climate Change**

America’s Climate Choices: Panel on Informing Effective Decisions and Actions Related to Climate Change

Washington, DC:National Academies Press, 2010. 300 pp. ISBN: 978-0-309-14594-7, $67

**Institutional Dynamics: Emergent Patterns in International Environmental Governance**

Oran R. Young

Cambridge, MA:MIT Press, 2010. 232 pp. ISBN: 978-0-262-01438-0, $48

**Machine Learning Approaches to Bioinformatics**

Zheng Rong Yang

Hackensack, NJ:World Scientific, 2010. 336 pp. ISBN: 978-981-4287-30-2, $107.00

**Principles of Environmental Sciences**

Jan J. Boersema, Lucas Reijnders, eds.

New York:Springer, 2010. 542 pp. ISBN: 978-90-481-9193-2, $79.85

**Sustainability Matters: Environmental Management in Asia**

Lin-Heng Lye, George Ofori, Lai Choo Malone-Lee, Victor R Savage, Yen-Peng Ting, eds.

Hackensack, NJ:World Scientific, 2010. 652 pp. ISBN: 978-981-4322-90-4, $138

**The Environmental Politics of Sacrifice**

Michael F. Maniates, John M. Meyer

Cambridge, MA:MIT Press, 2010. 344 pp. ISBN: 978-0-262-01436-6, $50

**from September**

**Towards a Liveable and Sustainable Urban Environment: Eco-Cities in East Asia**

Liang Fook Lye, Gang Chen, eds.

Hackensack, NJ:World Scientific, 2010, 232 pp. ISBN: 978-981-4287-76-0, $65

**Toxicity Pathway-Based Risk Assessment: Preparing for Paradigm Change**

Ellen Mantus, Rapporteur; Standing Committee on Risk Analysis Issues and Reviews; National Research Council

Washington, DC:National Academies Press, 2010. 134 pp. ISBN: 978-0-309-15422-2, $33.50

**Verifying Greenhouse Gas Emissions: Methods to Support International Climate Agreements**

Committee on Methods for Estimating Greenhouse Gas Emissions; National Research Council

Washington, DC:National Academies Press, 2010. 124 pp. ISBN: 978-0-309-15211-2, $30

**WHO Recommended Classification of Pesticides by Hazard and Guidelines to Classification 2009**

World Health Organization

Geneva:WHO Press, 2010. 78 pp. ISBN: 978-9-2415-4796-3, $30

**World Health Statistics 2010**

World Health Organization

Geneva:WHO Press, 2010. 177 pp. ISBN: 978-9-2415-6398-7, $40

**Progress on Sanitation and Drinking-Water: 2010 Update**

World Health Organization

Geneva:WHO Press, 2010. 54 pp. ISBN: 9789241563956, $20

**Scientific Basis of Tobacco Product Regulation**

Third Report of a WHO Study Group

Geneva:WHO Press, 2010. 50 pp. ISBN: 9789241209557, $15

**from July**

**Science: A Many-Splendored Thing**

Igor Novak

Hackensack, NJ:World Scientific, 2010. 200 pp. ISBN: 978-981-4304-74-0, $29

**Soviet Union in the Context of the Nobel Prize**

Abram M Blokh

Hackensack, NJ:World Scientific, 2010. 900 pp. ISBN: 978-981-4277-97-6, $98.00

**The Nature of Change or the Law of Unintended Consequences**

John Mansfield

Hackensack, NJ:World Scientific, 2010. 212 pp. ISBN: 978-1-84816-540-3, $45.0

Nobel Prizes and Life Sciences

Erling Norrby

Hackensack, NJ:World Scientific, 2010. 300 pp. ISBN: 978-981-4299-36-7, $68

**from May**

**Advances in Molecular Toxicology, 4**

James C. Fishbein

St. Louis, MO:Elsevier, 2010. 248 pp. ISBN: 978-0-444-53584-9, $244

**Understanding Climate’s Influence on Human Evolution**

Committee on the Earth system Context for Hominin Evolution; National Research Council

Washington, DC:National Academies Press, 2010. 128 pp. ISBN: 978-0-309-14838-2, $29.25

**Review of the Environmental Protection Agency’s Draft IRIS Assessment of Tetrachloroethylene**

Committee to Review EPA’s Toxicological Assessment of Tetrachloroethylene; National Research Council

Washington, DC:National Academies Press, 2010. 186 pp. ISBN: 978-0-309-15094-1, $42.25

**Comprehensive Toxicology, 2nd ed.**

Charlene A. McQueen

St. Louis, MO:Elsevier, 2010. 11,200 pp. ISBN: 978-0-08-046868-6, $5,195

**Verifying Greenhouse Gas Emissions: Methods to Support International Climate Agreements**

Committee on Methods for Estimating Greenhouse Gas Emissions; National Research Council

Washington, DC:National Academies Press, 2010. 144 pp. ISBN: 978-0-309-15211-2, $31.50

**Water Scarcity in the Mediterranean: Perspectives Under Global Change**

Sergi Sabater, Damià Barceló, eds.

New York:Springer, 2010. 232 pp. ISBN: 978-3-642-03970-6, $259

**from March**

**Choosing the Nation’s Fiscal Future**

Committee on the Fiscal Future of the United States; National Research Council and National Academy of Public Administration

Washington, DC:National Academies Press, 2010. 268 pp. ISBN: 978-0-309-14723-1, $53.95

**Evolution: The Modern Synthesis, The Definitive Edition**

Julian S. Huxley

Cambridge, MA:MIT Press, 2010. 784 pp. ISBN: 978-0-262-51366-1, $35

**Mismeasuring Our Lives**

Joseph E. Stiglitz, Amartya Sen, Jean-Paul Fitoussi

New York:New Press, 2010. 160 pp. ISBN: 978-1-59558-519-6, $15.95

**from January/February**

**Good Laboratory Practice Training Manual for the Trainee, 2nd ed.**

World Health Organization

Geneva, CH: WHO Press, 2009, 141 pp. ISBN: 9789241547574, $10.00

**Good Laboratory Practice Training Manual for the Trainer, 2nd ed.**

World Health Organization

Geneva, CH: WHO Press, 2009, 302 pp. ISBN: 9789241547567, $40.00

**2009**

**Science Research Writing for Non-Native Speakers of English**

Hilary Glasman-Deal

Hackensack, NJ:World Scientific, 2009. 272 pp. ISBN: 978-1-84816-309-6, $58

**The Year in Review 2008, Environment, Energy, and Resources Law**

Section of Environment, Energy, and Resources

Chicago:ABA Publishing, 2009. 379 pp. ISBN: 1946-9640, $75

**WHO Handbook on Indoor Radon**

World Health Organization

Geneva:WHO Press, 2009. 108 pp. ISBN: 978-9-2415-4767-3, $30

**Fundamentals of Patenting and Licensing for Scientists and Engineers**

Matthew Y Ma

Hackensack, NJ:World Scientific, 2009. 292 pp. ISBN: 978-981-283-420-1, $68

**Balancing Your Life: Executive Lessons for Work, Family and Self**

James G S Clawson

Hackensack, NJ:World Scientific, 2009. 368 pp. ISBN: 978-981-283-906-0, $35

**Communication Skills for the Biosciences**

Aysha Divan

New York:Oxford University Press, 2009. 288 pp. ISBN: 978-0-19-922635-1, $49.95

**Science: A Four Thousand Year History**

Patricia Fara

New York:Oxford University Press, 2009. 424 pp. ISBN: 978-0-19-922689-4, $34.95

**Solving Everyday Problems with the Scientific Method: Thinking Like a Scientist**

Don K. Mak, Angela T. Mak, Anthony B. Mak

Hackensack, NJ:World Scientific Publishing Co., 2009. 236 pp. ISBN: 978-981-283-509-3, $38

**Study & Communication Skills for the Biosciences**

Stuart Johnson, Jon Scott

New York:Oxford University Press, 2009. 248 pp. ISBN: 978-0-19-921983-4, $45

**The Manual of Scientific Style: A Guide for Authors, Editors, and Researchers**

Harold Rabinowitz, Suzanne Vogel

St. Louis, MO:Elsevier, 2009. 984 pp. ISBN: 978-0-12-373980-3, $54.95

**When the Scientist Presents: An Audio and Video Guide to Science Talks**

Jean-Luc Lebrun

Hackensack, NJ:World Scientific, 2009. 264 pp. ISBN: 978-981-283-919-0, $55

**Writing Scientific Research Articles: Strategy and Steps**

Margaret Cargill, Patrick O’Connor

Hoboken, NJ: John Wiley & Sons, Inc., 2009, 184 pp. ISBN: 978-1-4051-8619-3, $29.95

